# Genome analysis of *Mycobacterium avium* subspecies *hominissuis* strain 109

**DOI:** 10.1038/sdata.2018.277

**Published:** 2018-12-04

**Authors:** William M. Matern, Joel S. Bader, Petros C. Karakousis

**Affiliations:** 1 Department of Biomedical Engineering and High-Throughput Biology Center, Johns Hopkins University School of Medicine, Baltimore, MD, USA; 2 Center for Systems Approaches to Infectious Diseases, Johns Hopkins University School of Medicine, Baltimore, MD, USA; 3 Department of Medicine, Johns Hopkins University School of Medicine, Baltimore, MD, USA

**Keywords:** Bacterial infection, Gene ontology, Genome, Bacteriology, Software

## Abstract

Infection with *Mycobacterium avium* is a significant cause of morbidity and its treatment requires the use of multiple antibiotics for more than 12 months. In the current work, we provide the genome sequence, gene annotations, gene ontology annotations, and protein homology data for *M. avium* strain 109 (MAC109), which has been used extensively in preclinical studies. The *de novo* assembled genome consists of a circular chromosome of length 5,188,883 bp and two circular plasmids of sizes 147,100 bp and 16,516 bp. We have named the plasmids pMAC109a and pMAC109b, respectively. Based on its genome, we confirm that MAC109 should be classified as *Mycobacterium avium* subsp. *hominissuis*. Using genome annotation software, we identified 4,841 coding sequences and annotated these with Gene Ontology (GO) terms. Additionally, we wrote software to generate a database of homologous proteins among MAC109 and eight other commonly used mycobacterial laboratory strains. The resulting database may be useful for translating genetic data between various strains of mycobacteria, and the software may be applied readily to other organisms.

## Background & Summary

The group of environmental pathogens known as the nontuberculous mycobacteria is increasingly recognized as a significant cause of morbidity and mortality due to greater rates of immune suppression in the population, heightened awareness among clinicians of these infections, and improved diagnostic tests for mycobacterial species level identification^1–3^. *Mycobacterium avium* complex (MAC) frequently causes lung infection in elderly patients with chronic lung disease, as well as disseminated infection in patients with profound immune suppression including those with AIDS^4–6^.

Existing therapies for MAC infection are lengthy. Current guidelines for pulmonary infections recommend at least 1 year of therapy after culture conversion^7^, which has been estimated to occur after 5 months of therapy in most patients^8^. Additionally, an estimated 18% of MAC cases do not achieve culture conversion after one year^8^, suggesting therapy is substantially less effective in a large proportion of patients. Presently, little is known about the role of mycobacterial genes in the prolonged survival of *M. avium* in the antibiotic-treated host.

*M. avium* strain 109 (MAC109) was isolated from the blood of an AIDS patient and has been used frequently in preclinical investigations^9–13^. In our ongoing efforts to study the genes of *M. avium* via transposon mutagenesis (using ΦMycomarT7^14^), we observed the highest insertion efficiency with this strain of those we tested (MAC104, two colony morphotypes of MAC101, MAC3388, and MAC109). In an effort to enhance the potential utility of MAC109 for future studies focused on *M. avium* genetics, including RNA-seq and ChIP-seq analyses, we sequenced the MAC109 genome. Given the substantial genetic heterogeneity observed between *M. avium* isolates^15^ (such as the frequent inclusion of plasmids), we performed *de novo* assembly. We also provide Gene Ontology (GO) annotations, which have proven useful in exploratory analysis of the roles of individual genes.

The genomes of a few laboratory strains of mycobacteria have been extensively characterized (particularly the *M. tuberculosis* strain H37Rv and *M. smegmatis* strain mc^2^155). To facilitate the transfer of accumulated knowledge between mycobacterial strains, we computed a small database of protein homology scores between nine commonly studied strains (including MAC109) using exact global alignment software. Our hope is that this small database will be useful to investigators with expertise in specific proteins, which may have close homologs in other mycobacterial strains. This database also may be useful for prioritizing drug targets with potential for treating multiple mycobacterial diseases. Finally, our computer code may be applied for analyzing the genomes of other bacterial species.

## Methods

### Bacterial strain

MAC109 was received on agar as a gift from Dr. Luiz Bermudez (Oregon State University). A single colony was inoculated into Middlebrook 7H9 broth supplemented with 10% OADC, from which stocks were made. These stocks were frozen and subsequently passaged into fresh broth for generation of bacterial samples for DNA extraction.

### DNA Extraction for short read sequencing

DNA was extracted from MAC109 using two different methods. For Illumina sequencing we used a fast DNA extraction protocol that produces fragmented DNA. First, a 2 mL O-ring tube was filled with 1 gram of 0.1 mm zirconia/silica beads (Biospec #11079101z). Then 0.6 mL of liquid containing bacteria was transferred to each tube of beads. 2uL of RNase A (10 mg/mL, Thermo-Fisher #EN0531) was then added to each tube. Tubes were bead-beaten for 1 min at max speed. Tubes were then incubated at room temperature for 10 min to degrade RNA. 0.6 mL of saturated phenol solution was then added to tubes. Tubes were then briefly vortexed and spun for 2 min at 16,000 g. The aqueous phase was transferred to clean microcentrifuge tubes and an equal volume of chloroform/isoamyl (24:1, v/v) was added. Tubes were briefly vortexed and then centrifuged for 2 minutes at 16,000 g. 400 uL of the aqueous phase was then transferred to clean microcentrifuge tubes. 40 uL of 3 M sodium acetate was added and tubes were inverted to mix. 880uL of 100% ethanol was added and tubes were inverted to mix. If the DNA pellet was visible after mixing then tubes were spun at 16,000 g for 2 min, otherwise they were spun for 15 min. Supernatant was removed and 700 uL of 70% ethanol was added to wash the pellet. Tubes were centrifuged for an additional 30 s. Supernatant was removed and pellet was allowed to dry at room temperature for 15 min. Pellet was then dissolved in 100 uL of TE.

### DNA Extraction for long read sequencing

For PacBio sequencing, which requires high molecular weight DNA, bead beating could not be used, and a high molecular weight DNA extraction protocol for mycobacteria^16^ was adapted for this purpose. First, a bacterial pellet was obtained in a 2 mL Eppendorf tube in 500 uL TE buffer (pH 8.0). We then added an equal volume of chloroform/methanol (2:1, v/v) and rocked on a platform rocker for 5 min. Then we centrifuged the suspension at 16,000 g for 2 min. The aqueous and organic phases were removed by pipetting, leaving only the band of bacteria at the interface. The delipidated bacteria were then placed in a dry bath at 55 ^o^C for 10–15 min to remove traces of the organic phase. 550 uL of 10 mg/mL Lysozyme (Sigma-Aldrich, dissolved in TE buffer) was then added to the bacteria. Pellet was gently resuspended with a pipette. Tubes were then placed in a 37 ^o^C incubator overnight. On the following day 120 uL of 5% SDS (w/v) and 8uL proteinase K (10 mg/mL, Thermo-Fisher #EO0491) were added to the tubes. Tubes were then mixed by gentle inversion and incubated at 50 ^o^C for 3 hours in a dry bath. An equal volume (~ 750 uL) of PCI (phenol solution/chloroform/isoamylalcohol, 25:24:1, v/v) was then added and gently inverted on a rocker plate for 30 min. Tubes were then centrifuged at 16,000 g for 5 min. ~ 600 uL of the aqueous phase was then transferred to a clean microcentrifuge tube. 2 uL of RNase A (10 mg/mL, Thermo-Fisher #EN0531) was added and tubes were inverted to mix. Tubes were then incubated at room temperature for 10 min to remove contaminating RNA. Chloroform/isoamylalcohol (24:1, v/v) was then added and tubes were inverted for 30 s to mix thoroughly. Tubes were spun for 2 min at 16,000 g and 400uL of the aqueous phase was transferred to a clean microcentrifuge tube. 40 uL of 3 M NaCl was added and tubes were mixed by inversion. 0.8 mL of 100% EtOH was added and tubes were mixed by inversion. If DNA pellet was visible by eye then tubes were spun at 16,000 g for 2 min at room temperature, otherwise they were spun for 15 min. The supernatant was then removed from the pellet and 0.8 mL of 70% ethanol was added to wash the pellet. Tubes were then spun at 16,000 g for 5 min and supernatant discarded. DNA pellets were dried for 15 min at room temperature. After drying, pellets were resuspended in 100 uL TE buffer.

### Sequencing and *de novo* assembly of MAC109 genome

Library preparation and sequencing were performed by the Deep Sequencing and Microarray Core at the Johns Hopkins School of Medicine. Short reads were generated using an Illumina Miseq (2 × 75 bp). Library preparation was done with the Illumina TruSeq DNA Nano kit with target insert size of 350 bp. After clipping adapters (internally by Illumina software), there were 3,117,540 reads, which varied in size from 35 to 76 bp (472,180,593 total bases sequenced). Long reads were generated with a PacBio RSII after library preparation with the SMRTbell Template Preparation kit 1.0 and target read size of 10-20 kb. Raw PacBio data (*.h5) were converted to subreads (fastq format) with SMRT Tools v5.1.0 (bax2bam v0.0.8, bam2fastq v1.1.1). Raw reads from the Illumina Miseq and Pacbio RSII machines are available in the Sequence Read Archive (SRA) (Data Citation 1).

**Figure 1:**
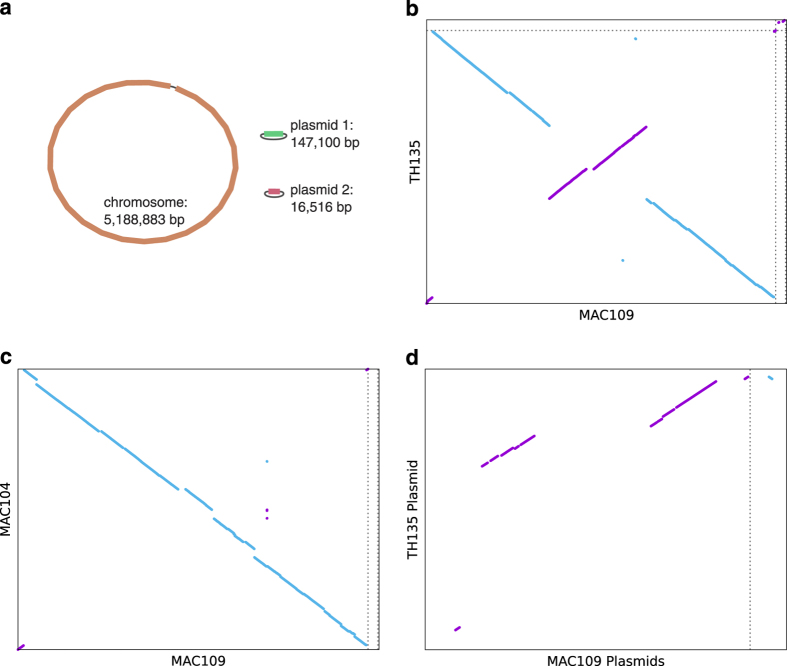
Summary of genomes assembly and homology. (**a**) MAC109 genome assembly containing 3 circular contigs. (**b**,**c**) Dot plots comparing TH135, MAC104, and MAC109 genomes assemblies. Dotted lines separate the replicons of each strain (TH135 has a single plasmid, MAC104 lacks plasmids). (**d**) Dot plot comparing the plasmid from the TH135 genome and the two plasmids from the MAC109 genome.

Reads smaller than 300 bp were filtered out using Trimmomatic v0.36^17^. After filtering, there were 151,792 subreads and 1,365,362,111 total bases sequenced. Assembly of the genome was performed using Unicycler pipeline v0.4.4 in conservative mode^18^. Dependencies for Unicycler were satisfied with SPAdes v3.11.1^19^, racon v1.2.1^20^, bowtie2 v2.3.4.1^21^, and pilon v1.22^22^. Unicycler was run in hybrid mode, allowing the use of both the Miseq (short, accurate) reads and PacBio (long, less accurate) reads. This produced an assembly with four circular contigs. However, one of these contigs was (nearly) identical to the genome of the bacteriophage PhiX174. It is highly unlikely that this phage is part of the MAC109 genome and much more likely that this contig was assembled from contaminating reads from the PhiX library run alongside our sample on the Miseq. The PhiX contig was therefore removed from the Unicycler assembly. Additionally, preliminary annotation revealed that the sequence of one of the plasmids started inside a gene. Since this complicates downstream processing, the start of the sequence was moved accordingly. Bandage v0.8.1^23^ was used to visualize the final genome assembly (Fig. 1a).

### Gene and Gene Ontology (GO) Annotations

Gene annotation was done with the Prokaryotic Genome Annotation Pipeline (PGAP) available from the National Center for Biotechnology Information^24^ (Data Citation 2). Proteins were given GO annotations using the PANNZER2 webservice with default settings^25^ (Gene Ontology Annotations for MAC109 Genome, Data Citation 3). We submitted our protein sequences for GO annotation on 2018/06/19 (the PANNZER2 databases are updated monthly).

### Genome Comparisons

The nucmer program from MUMmer v4.0.0beta2^26^ was used to compute SNPs and dot plots between the MAC104, TH135, and MAC109 genomes. Settings for the full genome dot plots (Fig. 1b and c) were “nucmer --maxmatch -l 20” followed by “delta-filter -l 2500 -m”. For dot plots comparing the TH135 and MAC109 plasmids, settings were “nucmer --maxmatch -l 10” followed by “delta-filter -l 1000 -m”. Plots were made with gnuplot v5.2 patchlevel 4 (Fig. 1b–d). To confirm the subspecies of MAC109 we also used nucmer (with settings: –maxmatch and –minmatch = 10) to check for the presence of insertion elements IS900, IS901, IS1311, and DT1.

### Homology database of common mycobacterial laboratory strains

We compared the annotated genes from several mycobacterial genomes commonly used for preclinical studies. We downloaded the annotations from Mycobrowser^27^ (where available) or GenBank for the following genomes: *M. avium hominissuis* strain 104 (ftp://ftp.ncbi.nlm.nih.gov/genomes/all/GCA/000/014/985/GCA_000014985.1_ASM1498v1), *M. avium hominissuis* TH135 (ftp://ftp.ncbi.nlm.nih.gov/genomes/all/GCA/000/829/075/GCA_000829075.1_ASM82907v1), *M. avium paratuberculosis* K10 (ftp://ftp.ncbi.nlm.nih.gov/genomes/all/GCA/000/007/865/GCA_000007865.1_ASM786v1), *M. marinum* strain M also called ATCC BAA-535 (https://mycobrowser.epfl.ch/releases/3/get_file?dir=fasta_proteins&file=Mycobacterium_marinum_M.fasta), *M. tuberculosis* H37Rv (https://mycobrowser.epfl.ch/releases/3/get_file?dir=fasta_proteins&file=Mycobacterium_tuberculosis_H37Rv.fasta), *M. smegmatis* mc^2^155 (https://mycobrowser.epfl.ch/releases/3/get_file?dir=fasta_proteins&file=Mycobacterium_smegmatis_MC2-155.fasta), *M. abscessus* ATCC 19977 (https://mycobrowser.epfl.ch/releases/3/get_file?dir=fasta_proteins&file=Mycobacterium_abscessus_ATCC_19977.fasta), *M. intracellulare* ATCC 13950 (ftp://ftp.ncbi.nlm.nih.gov/genomes/all/GCA/000/277/125/GCA_000277125.1_ASM27712v1).

For each pair of genomes this comparison was done in an “all-vs.-all” fashion, wherein each protein-coding gene in genome A was aligned to every coding gene of genome B (pseudogenes and RNA genes were excluded from this analysis). Alignment was done using the Needleman-Wunsch (NW) exact global alignment algorithm. Global alignment was chosen (over local alignment), as our goal was to identify genes with similar functions (i.e., biochemical activities). We assume that genes with large amino acid differences at the ends are likely to have some distinct functions. Exact alignment was chosen over heuristic-based alignment (such as BLAST^28^) to improve sensitivity at the expense of speed. Dependencies for our scripts include BioPython^29^ (for sequence processing), opal^30^ (fast NW implementation), and parasail^31^ (another NW implementation). Opal was found to be significantly faster than parasail but produced less detailed output (e.g., opal did not report percent identity for each comparison). Therefore, we used opal to quickly remove all but a few candidate matches, which were then checked by parasail.

The score settings used for the final NW alignment by parasail were: 2 for matches, 0 for mismatches, −20 for opening a gap, −2 for extending a gap. We used a single value for scoring all amino acid matches because our goal was to identify proteins with large sequences of identical matches rather than simply provide evidence of homology (for which BLOSUM or PAM scoring matrices would be more appropriate). Percent identities between genes were calculated with parasail as the number of identical amino-acids divided by the length of the query sequence.

Candidate matches were generated with opal using NW score settings: 1 for matches, 0 for mismatches, 0 for opening a gap, and 0 for extending a gap. A strict upper bound for the final percent identity (computed by parasail) could then be calculated by dividing the opal score by the length of the query. Matches below 50% identity could therefore be guaranteed to be below the minimum 50% percent identity threshold required in the final alignment and were removed before passing to parasail.

### Code availability

Scripts to compute alignments and percent identities between proteomes are available online^32^ along with instructions for use (see above for URLs to mycobacterial genomes/proteomes used in this project).

## Data Records

### Data Record 1

The genome of MAC109 consists of a circular chromosome (5,188,883 bp) and two plasmids of sizes 147,100 bp and 16,516 bp (Fig. 1a), and approximate multiplicities (estimated by Unicycler) of 1.76x, 4.92x, respectively. We have named the larger plasmid pMAC109a and the smaller plasmid pMAC109b. Based on the presence of insertion element IS1311 (GenBank accession no. U16276) and absence of IS900 (accession no. X16293), IS901 (accession no. X59272), and DT1 (accession no. L04543) we confirm that MAC109 belongs to the avium subspecies *hominissuis*^33^. PGAP identified 4,841 protein coding sequences, 53 RNA genes (including 46 tRNAs, 4 rRNAs, and 3 ncRNAs), and 191 pseudogenes. The annotated genome and raw reads are available under GenBank accession numbers CP029332-CP029334 (Data Citation 2).

We compared the MAC109 genome to those of TH135 and MAC104. MUMmer/nucmer estimated 32,974 SNPs relative to MAC104 and 56,751 SNPs relative to TH135 (there were 46,685 SNPs between TH135 and MAC104). Dot plots of these comparisons are presented in Fig. 1b–d. Notably, a number of large-scale inversion have occurred in these strains. Additionally, the pMAC109a plasmid from the MAC109 assembly shares significant regions of similarity with the plasmid in TH135, although there are large distinct regions as well (Fig. 1d).

### Data Record 2

PANNZER2 provided a total of 17,292 GO annotations for 3860 unique proteins (among a total of 4841 proteins) (Gene Ontology Annotations for MAC109 Genome, Data Citation 3). The full list and descriptions of the GO terms can be found at http://www.geneontology.org/page/download-ontology.

### Data Record 3

We provide three tables of our mycobacterial homology database with different percent identity cutoffs. Each row of the table was calculated by querying a particular protein (name is provided in the second column) against the proteins of each mycobacterial genome in the database (genome names listed in the first row). All matches exceeding the threshold (percent identity > 50%, > 75%, or > 90%) within each genome are listed in each element of the respective table. The percent identity is listed in parentheses next to each match. Both orthologs (i.e., matches between genomes) and paralogs (i.e., matches within the same genome) are reported (Mycobacterial Homology Database with 50% Identity Cutoff, Data Citation 3).

## Technical Validation

To provide support for our assembled MAC109 genome we show that using an entirely different assembler (Canu v1.7.1) yields an almost identical genome thus supporting our reported genome. However, unlike Unicycler, Canu is not a hybrid assembler and does not attempt to circularize contigs. Therefore, a few minor differences are expected. In particular, the assembled contigs are linear and might have identical regions at the ends of contigs when the underlying DNA molecules are circular. Also, Canu does not attempt to move the origin of circular contigs after assembly. Therefore, the Canu-derived contigs are likely to have different origins than the Unicycler contigs.

**Figure 2:**
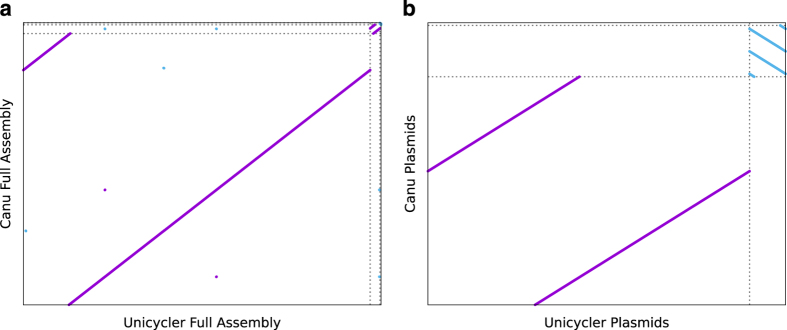
Comparison of genome assembly of MAC109 output by Canu vs Unicycler. (**a**) Dot plot comparing full Unicycler (3 contigs) assembly vs full Canu assembly (4 contigs). Dotted lines separate the replicons of each strain. (**b**) Same comparison but with the largest contig from each assembly removed. This provides a higher resolution comparison of the small contigs.

After running (“canu genomeSize = 5.35 m -pacbio-raw pb_reads.fastq.gz”, other settings set to defaults), Canu assembled 4 linear contigs (Lengths: 5,207,511 bp, 167,345 bp, 37,619 bp, and 1974bp). To compare the Canu assembly with the Unicycler assembly we used MUMmer (“nucmer –maxmatch -l 20” followed by delta-filter to filter out small matches). Fig. 2a shows a dotplot comparing the entire genomes (“delta-filter -l 2500”). Figure 2b compares the smaller contigs (plasmids) of the Unicyler and Canu assemblies (“delta-filter -l 1000”). This shows that the Canu contigs repeat themselves at the ends (and in the case of contig 3, repeats occur multiple times), as expected. Secondly, it can be seen that contigs 1-3 are nearly identical to the Unicycler contigs, supporting the accuracy of our assembly.

However, Canu assembled one additional contig (Canu contig 4, length = 1974bp) relative to Unicycler. Canu contig 4 was noted to have a high TA content, although mycobacteria are known to be GC-rich. To test whether the Canu contigs are actually present in the MAC109 genome, we mapped our collected Illumina reads (Data Citation 1), which were not used in the assembly by Canu, to estimate the multiplicity of each contig. We used bowtie2 v2.3.4.1 with default settings. Overall, > 97.9% of the Illumina reads mapped to the Canu contigs. We then used samtools v1.5 (“samtools sort -o file2.bam file1.sam” followed by “samtools depth file2.bam”) to calculate the depth at each position and averaged the depth across each contig to calculate the coverage using a short python3 script. Coverage was 83x for contig 1, 128x for contig 2, and 177x for contig 3. 0 reads mapped to the 1974bp contig output by Canu. Therefore, contig 4 appears to be an entirely erroneous contig output by Canu.

We have shown that our assembled genome is robust to changes in the assembler, although Canu produces an erroneous contig. This supports the quality of our genome sequence for MAC109 (Data Citation 2).

## Usage Notes

We have provided the complete genome sequence and gene annotations, along with gene ontology annotations for MAC109. We hope these data will enable detailed genetic studies of this and other strains of *M. avium*. For example, our current work is focused on identifying the essential and non-essential genes of *M. avium* through saturating transposon mutagenesis. The availability of a high-quality genome greatly simplifies the analysis involved in this effort. As additional knowledge of mycobacterial strains accumulates, the ability to transfer protein information from one strain to another may reduce costs and effort. Our mycobacterial homology database (and scripts to extend it to other strains) may be useful for this purpose.

The provided genome and homology database (Data Citation 3) complements the existing Mycobrowser database and tuberculosis database (TBDB). First, our provided database includes strains not included in Mycobrowser (e.g., the *M. avium* and *intracellulare* genomes) and can be easily extended to any other genome of interest with the provided software. Second, Mycobrowser identifies homologs between organisms by first identifying homologs in *M. tuberculosis* H37Rv. As a consequence, homologs are sometimes missed. For example, the rifampin ADP-ribosyl transferase in *M. smegmatis* mc^2^155 (MSMEG_1221) has no listed homologs despite the existence of a homolog in *M. abscessus* ATCC_19977 (MAB_0591). Thus, our provided database can be more sensitive for these. Lastly, we have designed our database to be simple to use for both quick manual lookups as well as genome-scale mappings between organisms. Thus, we have packaged the database to be both human-readable and easily parsed by a computer. In particular, we find our table format somewhat easier to use for these purposes than the SYNERGY output available from TBDB (http://www.broadinstitute.org/ftp/pub/seq/msc/pub/SYNERGY/synergy_final31.txt).

We hope the items provided with this data descriptor will accelerate research into *M. avium* and support the development of novel therapeutics to prevent and treat human diseases caused by these pathogens.

## Additional information

**How to cite this article**: Matern, W. M. *et al*. Genome analysis of *Mycobacterium avium* subspecies *hominissuis* strain 109. *Sci. Data*. 5:180277 doi: 10.1038/sdata.2018.277 (2018).

**Publisher’s note**: Springer Nature remains neutral with regard to jurisdictional claims in published maps and institutional affiliations.

## Supplementary Material
